# Increased risk of infections in smoldering multiple myeloma: results from the screened iStopMM study

**DOI:** 10.1038/s41375-025-02762-9

**Published:** 2025-09-12

**Authors:** Lærke Sloth Andersen, Ricardo Berenguer Navarro, Sara Ekberg, Sæmundur Rögnvaldsson, Marína Rós Levy, Ingigerður Sólveig Sverrisdóttir, Brynjar Viðarsson, Páll Torfi Önundarson, Bjarni A. Agnarsson, Margrét Sigurðardóttir, Ingunn Þorsteinsdóttir, Ísleifur Ólafsson, Ásdís Rósa Þórðardóttir, Elías Eyþórsson, Ásbjörn Jónsson, Andri Ólafsson, Brian G. M. Durie, Stephen Harding, Ola Landgren, Thorvardur Jón Löve, Sigurður Yngvi Kristinsson, Sigrún Thorsteinsdóttir

**Affiliations:** 1 Department of Hematology, Rigshospitalet, Copenhagen, Denmark; 2 Red Door Analytics AB, Stockholm, Sweden; 3 University of Iceland, Reykjavik, Iceland; 4 Landspitali, Reykjavik, Iceland; 5 Akureyri Hospital, Akureyri, Iceland; 6 Samuel Oschin Comprehensive Cancer Institute, Cedars-Sinai Outpatient Cancer Center, Los Angeles, CA USA; 7 Binding Site Group Ltd, Birmingham, UK; 8 University of Miami, Sylvester Comprehensive Cancer Center, Miami, FL USA

**Keywords:** Myeloma, Myeloma, Epidemiology

## Abstract

Infections are a major cause of morbidity and mortality in multiple myeloma (MM). While increased infection risk has been shown in monoclonal gammopathy of undetermined significance (MGUS), data are limited for smoldering multiple myeloma (SMM). We used data from the iStopMM study, which screened 75,422 Icelandic individuals aged ≥40 years for MM precursors. Individuals diagnosed with SMM were matched by age and sex with MGUS-free comparators (1:5 ratio) and with individuals with MGUS (1:1 ratio). Infection outcomes were derived from nationwide registries of ICD-10 diagnostic codes and antimicrobial prescriptions. Cox proportional hazards models estimated infection risk, adjusted for immunoparesis. 188 SMM individuals were matched to 188 MGUS individuals and 162 SMM individuals were matched to 810 comparators. Individuals with SMM had significantly more infections (HR 1.36, 95% CI 1.07–1.73) and antibacterial prescriptions (HR 1.24, 95% CI 1.01–1.52) than the comparators. Compared to MGUS, individuals with SMM also had more infections (HR 1.37, 95% CI 1.00–1.87). Adjusting for immunoparesis attenuated the associations, suggesting it may partially mediate infection risk. This first screened cohort of SMM shows a significantly increased infection risk, compared to both MGUS and to individuals without MM precursors, suggesting an underrecognized infection burden in SMM.

## Introduction

Multiple myeloma (MM) is a plasma cell malignancy and the second most common hematologic cancer. MM almost always evolves from the asymptomatic precursor, monoclonal gammopathy of undetermined significance (MGUS) [1]. The more advanced precursor condition, smoldering multiple myeloma (SMM), represents an intermediary disease state between MGUS and MM, and is defined by monoclonal bone marrow plasma cells (BMPC) > 10% and/or an M-protein level ≥30 g/L, without evidence of end-organ damage i.e., CRAB criteria or other myeloma defining events [2]. We previously described the epidemiological and clinical characteristics of SMM in the general population and found that the prevalence of SMM is 0.5% in persons above 40 years [3].

Infections are a major cause of morbidity and mortality in MM and previous studies have shown that patients with MM have a significantly increased risk of infections compared to the general population. As new treatments for MM have improved survival rates, effective infection management and prevention become increasingly important [4]. A recent Swedish population-based study reported a 5-fold increased risk of infections in MM patients compared to controls and interestingly, found elevated infection rates beginning up to four years before MM diagnosis [5]. Similarly, a recent Danish study observed rising antimicrobial prescriptions up to a decade before MM diagnosis, with a marked increase in the last 5 years preceding diagnosis [6]. These findings raise the question of whether infection risk is confined to those who eventually progress to MM or extends to all individuals with MM precursors.

An increased risk of infections has been observed in MGUS, a precursor condition that may precede MM by decades. However, these studies are based on clinical cohorts and are biased by underlying comorbidities [7, 8]. Given that SMM represents a more advanced stage of clonal plasma cell proliferation than MGUS, it is reasonable to hypothesize that the infection burden could be even greater.

While infection risk is well-documented in MM, little is known about its burden in SMM, and to our knowledge, the infection risk in individuals with SMM has not previously been investigated. As treatment strategies for SMM are increasingly discussed, some recommending early intervention, a deeper understanding of baseline infection risk is essential and could inform future preventive strategies and optimize patient management [9, 10]. Understanding the infection burden in SMM is not only important for supportive care but could also provide insight into disease biology and help identify patients at higher risk of progression.

The aim of this study was therefore to investigate the risk of infection in individuals with SMM compared to individuals with MGUS and individuals without MM precursors in the large Iceland Screens, Treats, or Prevents Multiple Myeloma study (iStopMM).

## Methods

### Study cohorts

In this matched cohort study, we used data from the iStopMM study, a population-based screening study for MM precursors in Iceland where a total of 75 422 persons ≥40 years old were screened using serum protein electrophoresis (SPEP) and a free light chain (FLC) as well as an immunoglobulin assay from 2016-2020. Individuals with detected M-protein or abnormal FLC results were randomized into three arms. In arm 1 the individuals continued standard care in the Icelandic healthcare system. Individuals in arm 2 were followed according to current guidelines and individuals in arm 3 received a more intensive follow-up where bone marrow sampling was offered to everyone. Initial assessment and follow-up for individuals in arm 2 and 3 was performed at the iStopMM study clinic. Through systematic bone marrow sampling, individuals with bone marrow plasma cells between 10-60% and no myeloma defining events were diagnosed with SMM. Likewise, individuals with less than 10% bone marrow plasma cells, an M-protein level below 30 g/L and no myeloma defining events were diagnosed with MGUS [11].

Included were all individuals diagnosed with SMM and matched individuals with MGUS and comparators without MGUS, defined as individuals with a negative screening result. Individuals with SMM were matched 1:5 to MGUS-free comparators based on sex, age (5-year groups), and calendar month of diagnosis/screening, and 1:1 to MGUS individuals, additionally matched on study arm. The diagnosis date for individuals with SMM or MGUS was defined as the date of the relevant study clinic visit, and MGUS-free comparators were matched based on their screening date. Matching within study arm ensured access to updated disease status, allowing inclusion of all SMM individuals in the MGUS comparison. In contrast, only SMM individuals diagnosed within the screening period could be included in the comparison with MGUS-free comparators. Individuals who had already received a diagnosis of MGUS or lymphoma before screening or who had missing immunoglobulin data were excluded from the analysis. Individuals with SMM were classified into three groups based on the Mayo Clinic’s 2-20-20 risk stratification model: low-, intermediate-, and high-risk SMM [12].

In Iceland, there are no national vaccination guidelines specifically for individuals with MGUS or SMM, but clinical practice is to follow national recommendations for the general population. These include annual influenza vaccinations, and pneumococcal vaccinations and COVID-19 vaccines for those over 60 years or with relevant comorbidities. Thus, the three groups involved in the study follow the same recommendations and practices regarding vaccinations.

### Study outcomes

Outcomes were defined as infections or antimicrobial prescriptions according to either ICD-10 codes or ATC codes acquired from centralized registries encompassing all medical care in Iceland (supplementary Tables 1–4). The Register of Primary Health Care Contacts and the Hospital Discharge Register include all primary care and outpatient visits as well as inpatient admissions. Information on antimicrobial use is available from the Icelandic Prescription Medicines Registry which includes all prescriptions in Iceland from 2002.

Infections were analyzed overall, and in the subgroups, bacterial, viral, fungal, and other infections. Although parasitic infections were considered in overall infections, a separate analysis for parasitic infections exclusively was not performed due to few events. Antimicrobials were classified into overall, antibacterial, antiviral, and antifungal medications. Chronic infections e.g., Human Immunodeficiency Virus and Hepatitis C Virus as well as their appertaining antimicrobials were excluded. When considering more than one infection all events with the same ICD-10 or ATC code recorded within 30 days of one another were assumed to be the same episode and thus counted only once. Follow-up started on date of SMM or MGUS diagnosis, or date of screening for comparators and ended on date of death, disease progression, start of treatment for their plasma cell disorder including inclusion to the iStopMM treatment trial, or May 1st, 2022.

### Statistical analyses

Two separate analyses were conducted: one for time-to-first infection and one for recurrent infections. For the time-to-first infection analysis, adjusted Cox proportional hazard models were used to estimate hazard ratios (HRs) with 95% confidence intervals (CIs). In the recurrent infection analysis, a frailty Cox proportional hazards model was applied to account for the possibility of multiple infections per individual during follow-up. In this model, a random effect (frailty term) was included to account for within-individual correlation.

The proportional hazards assumption was tested using Schoenfeld residuals. All models were adjusted for age and sex. A sensitivity analysis adjusting for immunoparesis was also conducted, where immunoparesis was defined as the suppression of at least one uninvolved immunoglobulin below normal reference range.

## Results

A total of 188 individuals with SMM were included in the comparison with MGUS (1:1 matched within study arm to ensure up-to-date diagnostic status). Of these, 162 were diagnosed during the screening period and were matched to five MGUS-free comparators each (N = 810). The baseline characteristics of individuals with SMM included in the study are presented in Table 1.

**Table 1:** Baseline characteristics of individuals with smoldering multiple myeloma and their matched MGUS-free comparators and matched MGUS individuals.

	SMM	MGUS-free comparators	MGUS
Total, *N*	188	810	188
Age (years), median (IQR)	69 (63–76)	69 (62–76)	69 (63–77)
Sex, N (%)			
Female	73 (39%)	310 (38%)	73 (39%)
Male	115 (61%)	500 (62%)	115 (61%)
Follow-up (years), median (IQR)	2.16 (1.26–4.02)	3.73 (2.26–4.32)	2.72 (1.37–4.06)
M-protein isotype, N (%)			
Biclonal	18 (9.5%)	-	13 (7%)
IgA	45 (24%)	-	9 (5%)
IgG	97 (51.5%)	-	114 (60%)
IgM	2 (1%)	-	28 (15%)
Light-chain MGUS	26 (14%)	-	24 (13%)
M-protein level (g/L), median (IQR)	5.9 (3.3–10.7)	-	2.2 (1.4–3.7)
Hemoglobin (g/L), median (IQR)	138 (128–147)	142 (133–151)	141 (131–149)
Total calcium (mmol/L), median (IQR)	2.37 (2.31–2.43)	2.36 (2.29–2.41)	2.37 (2.30–2.43)
Creatinine (µmol/L), median (IQR)	87 (76-101)	84 (70–96)	86 (74–104)
Immunoparesis, N (%)			
1 uninvolved Ig < normal ref.	86 (46%)	77 (9.5%)	42 (22%)
25% reduction from normal ref. in 1 Ig	51 (27%)	24 (3.0%)	19 (10%)
>1 Ig under normal ref.	32 (17%)	7 (0.9%)	10 (5.3%)
Risk factors (2-20-20 model), N (%)			
M-protein >2 g/L	3 (1.6%)	-	-
FLC ratio >20 or <0.05	21 (11%)	-	-
BMPC > 20%	28 (15%)	-	-
SMM risk stratification, N (%)			
0 risk factors: Low risk	143 (76%)	-	-
1 risk factor: Intermediate risk	39 (21%)	-	-
2-3 risk factors: High risk	6 (3%)	-	-

Data presented as medians with interquartile range (IQR).

*SMM* smoldering multiple myeloma, *MGUS* monoclonal gammopathy of undetermined significance, *Ig* immunoglobulin, *FLC ratio* free light chain ratio, *BMPC* bone marrow plasma cell concentration.

### Comparison of SMM and MGUS free comparators

#### Baseline characteristics

The 162 individuals with SMM and 810 matched comparators had a median age of 69 years, with 62% men in both groups. Immunoparesis was more common among individuals with SMM than comparators (46% vs. 10%). The median follow-up time was 3 years for the SMM group and 3.7 years for the comparator group. Based on the 2-20-20 risk stratification model, 119 (73.5%) individuals were classified as low-risk, 37 (22.8%) as intermediate, and 6 (3.7%) as high-risk SMM. During follow-up, 6 (3.7%) individuals in the SMM group progressed to MM, while 40 (24.7%) were enrolled in a treatment trial for SMM. There were 6 (3.7%) deaths in the SMM group and 55 (6.8%) deaths among comparators.

#### Incidence and risk of infections

**Fig. 1:**
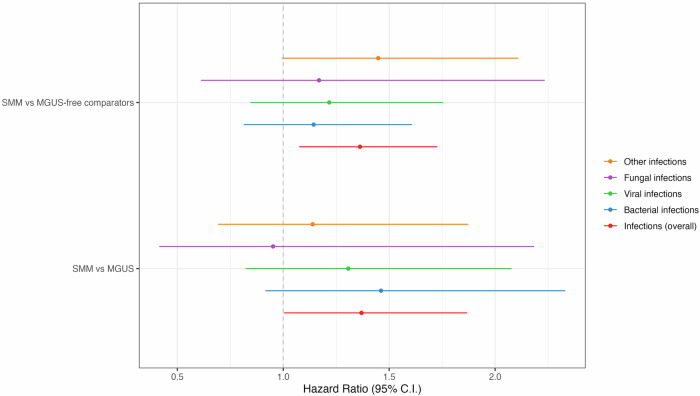
Time-to-first infection analysis. Forest plot of hazard ratios (HRs) with 95% confidence intervals (CIs) of subgroups of infections defined by ICD codes in individuals with SMM compared to MGUS-free comparators (top) and individuals with MGUS (bottom), estimates can be found in Tables 2 and 3 respectively.

**Fig. 2:**
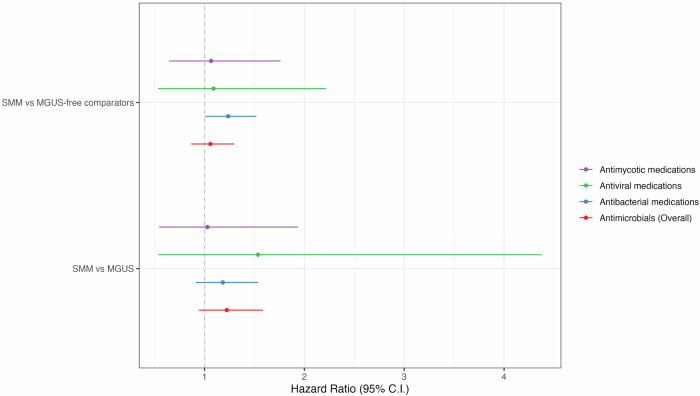
Time-to-first infection analysis. Forest plot of hazard ratios (HRs) with 95% confidence intervals (CIs) of subgroups of antimicrobials defined by ATC codes in individuals with SMM compared to MGUS-free comparators (top) and individuals with MGUS (bottom), estimates can be found in Tables 2 and 3 respectively.

Table 2 shows the number of diagnosed infections and antimicrobial prescriptions in each group along with hazard ratios (HRs) and 95% confidence intervals (CIs) for SMM compared to MGUS-free comparators. Overall, the SMM group had 181 infections, and 454 antimicrobial prescriptions registered during the study period, with 52.5% of the individuals having at least one infection and 71% having at least one antimicrobial prescription. In the comparator group, a total of 873 infections and 2472 antimicrobial prescriptions were registered, with 46.5% of the individuals having at least one infection and 71.9% having at least one antimicrobial prescription. In the time to first infection analysis, individuals with SMM had a significantly higher risk of overall infections (HR 1.36; 95% CI: 1.07-1.73). Estimates for bacterial, viral, fungal, and other infections were all numerically higher for the SMM group, although the differences were not statistically significant (Fig. 1). There was a higher rate of prescriptions of antibacterial medication in the SMM group compared to comparators with an HR of 1.24 (95% CI: 1.01-1.52) but no significant association was found for antiviral or antifungal prescriptions (Fig. 2). No significant associations were found for either infections or antimicrobials in the recurrent infection analysis although the estimates were higher for SMM (Table 2).

**Table 2:** Number of infections and antimicrobial prescriptions among SMM individuals and MGUS-free comparators.

	SMM	MGUS-free comparators	HR (95% CI)	
Total, N	162	810	Time-to-first infection	Recurrent infection
**All infections (ICD-10 codes), N**	181	873	**1.36 (1.07–1.73)**	1.22 (0.97–1.53)
Other infections, *N*	47	214	1.45 (0.99–2.11)	1.33 (0.89–2.01)
Bacterial infections, *N*	60	333	1.13 (0.80–1.59)	1.07 (0.75–1.51)
Viral infections, *N*	50	255	1.22 (0.85–1.75)	1.19 (0.82–1.73)
Fungal infections, *N*	24	71	1.14 (0.60–2.17)	1.37 (0.62–3.04)
**All antimicrobials (ATC codes), N**	454	2472	1.06 (0.87–1.30)	1.09 (0.91–1.31)
Antibacterial, *N*	413	2180	**1.24 (1.01–1.52)**	1.10 (0.91–1.32)
Antiviral, *N*	13	83	1.09 (0.54–2.22)	1.00 (0.48–2.08)
Antifungal, N	28	209	1.07 (0.65–1.76)	0.91 (0.52–1.60)

Relative risk of infections and antimicrobial prescriptions in SMM compared to matched comparators.

Data presented as total numbers of recurrences of the specific infection or antimicrobial. Relative risk presented as hazard ratios (HR) adjusted for age and sex with 95% confidence intervals for the time-to-first infection and recurrent infection analyses. Significant results in bold.

The most common infections and antimicrobials are listed in Supplementary Table 5. The most common infections in the SMM group were acute bronchitis, dermatophytosis, unspecified viral infection, acute sinusitis, and urinary tract infection (UTI). The most common bacterial infections were acute sinusitis, UTI and acute lower respiratory infection, and the most common viral infections included acute bronchitis, unspecified viral infection, Coronavirus Disease 2019, and herpes zoster. The pattern was largely similar in comparators, with respiratory infections and UTIs being the most frequent in both groups. This was reflected in the most frequently prescribed antimicrobials. For both groups, the most frequently prescribed antibacterials were amoxicillin or amoxicillin/clavulanic acid for SMM and comparators respectively, while valaciclovir and oseltamivir were the most frequently prescribed antivirals in both groups. A Cox proportional hazards analysis for specific classes of antibacterials showed the highest HRs for lincosamides (HR 1.55, 95% CI: 0.70–3.40), tetracyclines (HR 1.35, 95% CI: 0.87**–**2.09), and trimethoprim-sulfamethoxazole (HR 1.56, 95% CI: 0.90–2.71), though there were no significant associations. When adjusting for immunoparesis in the time-to-first infection analysis, the difference in risk of overall infections was smaller and no longer statistically significant (HR 1.26, 95% CI 0.98**–**1.63) and the difference in antibacterial prescriptions was not significant (HR 1.16, 95% CI: 0.94**–**1.44).

### Comparison of SMM and MGUS

#### Baseline characteristics

188 individuals with SMM and 188 matched individuals with MGUS were included with a median age of 69 years and a gender distribution of 61% men in both groups. 46% of the individuals in the SMM group had immunoparesis compared to 22% in the MGUS group. For the SMM cases the median follow-up time was 2.16 years and for the matched MGUS individuals it was 2.72 years. During the follow-up period, 5 (2.7%) individuals in the SMM group and 3 (1.6%) in the MGUS group progressed to MM, and 36 (19.1%) of the SMM individuals were enrolled in a treatment trial for SMM. In total there were 6 (3.2%) deaths in the SMM group and 16 (8.5%) deaths in the MGUS group.

#### Incidence and risk of infections

Table 3 shows the number of all infections and prescriptions in each group as well as the relative risk of infections and antimicrobials compared to comparators presented as HRs with 95% CI. Overall, the SMM group had 179 infections, and 448 antimicrobial prescriptions registered during the study period, with 46.8% of the individuals having at least one infection and 63.8% having at least one antimicrobial prescription. In the MGUS group, a total of 168 infections and 502 antimicrobial prescriptions were registered, with 41.5% of the individuals having at least one infection and 60.6% having at least one antimicrobial prescription. In the time to first infection analysis, individuals with SMM had an increased risk of overall infections (HR 1.37; 95% CI: 1.00**–**1.87) and the estimates for the risk of bacterial, viral, and other infections were increased in SMM, although not statistically significant (Fig. 1). No significant association was found for any antimicrobials (Fig. 2). In the recurrent infection analysis, no significant associations were found for either infections or antimicrobials (Table 3).

**Table 3:** Number of infections (ICD-10 codes) and antimicrobial prescriptions (ATC-codes) among SMM individuals and MGUS individuals.

	**SMM**	**MGUS**	**HR (95% CI)**	
Total, N	188	188	Time-to-first infection	Recurrent infection
**All infections (ICD-10 codes), N**	179	168	**1.37 (1.00–1.87)**	1.23 (0.93**–**1.63)
Other infections, *N*	43	47	1.14 (0.69**–**1.87)	1.02 (0.63**–**1.65)
Bacterial infections, *N*	60	57	1.46 (0.92**–**2.33)	1.23 (0.76**–**1.99)
Viral infections, *N*	53	49	1.31 (0.82**–**2.08)	1.24 (0.80**–**1.90)
Fungal infections, *N*	23	15	0.95 (0.42**–**2.18)	1.16 (0.34**–**3.98)
**All antimicrobials (ATC codes), N**	448	502	1.22 (0.94**–**1.59)	1.01 (0.78**–**1.30)
Antibacterial, *N*	406	432	1.18 (0.91**–**1.54)	1.95 (0.83**–**1.32)
Antiviral, *N*	13	36	1.54 (0.54**–**4.38)	0.99 (0.35**–**2.78)
Antifungal, *N*	29	34	1.03 (0.55**–**1.94)	0.93 (0.46**–**1.90)

Relative risk of infections and antimicrobial prescriptions in SMM compared to matched MGUS individuals.

Data presented as total numbers of recurrences of the specific infection or antimicrobial. Relative risk presented as hazard ratios (HR) adjusted for age and sex with 95% confidence intervals for the time-to-first infection and recurrent infection analyses. Significant results in bold.

The most common infections and antimicrobials overall and by subtypes are listed in Supplementary Table 6 for SMM and MGUS separately. The overall infection pattern was similar between SMM and MGUS individuals with respiratory, and to a lower extent urinary tract infections, being the predominant infections which reflected in the most common antimicrobials with amoxicillin being the most frequently prescribed antimicrobial for both groups. Adjusting for immunoparesis in the time-to-first infection analysis, did not change the estimate of the difference in risk of overall infections but the difference was not statistically significant (HR 1.37, 95% CI: 0.99-1.88).

## Discussion

In this population-based study including a screened SMM cohort, the infection risk in a SMM population was assessed for the first time and we found that individuals with SMM had an increased risk of infections compared to both MGUS and MGUS-free comparators. They also received more antibacterial prescriptions than comparators without MGUS. Interestingly, the study demonstrates a significant increase in infections even within a primarily low-risk SMM cohort. This is notable given that the majority of SMM individuals with higher-risk features—who might be more predisposed to infections—were censored early due to initiation of therapy as part of the iStopMM treatment trial. Taken together, this suggest that even a modest disease burden, such as having ≥10% bone marrow plasma cells, may confer an impaired immune function or other biological vulnerabilities contributing to an increased susceptibility to infections.

The overall infection patterns were similar among individuals with SMM, MGUS, and MGUS-free comparators, with respiratory infections being the most common across all groups. However, individuals with SMM experienced a relatively higher number of infections, suggesting a potential greater infectious burden despite a similar infection profile. Notably, the significantly higher rate of antibacterial prescriptions in the SMM group supports the interpretation that the infectious burden may be driven, at least in part, by bacterial infections or clinical suspicion thereof. However, the clinical relevance of these observations remains uncertain, as we lack detailed information on the severity of individual infections and whether they resulted in hospitalization. Moreover, the observed increase in infections must be interpreted in the context of the overall low absolute mortality in the SMM cohort.

The estimates of infection risk for both SMM vs comparators and SMM vs MGUS are very similar which might suggest that the MGUS individuals in this cohort are not at a high risk of infections in oppose to what previous studies have reported. This is likely attributable to the fact that MGUS cohort in this study was diagnosed through population screening and exhibits a less comorbid group of MGUS compared to clinically ascertained MGUS cohorts used in previous studies [7]. Infection risk in the screened iStopMM MGUS cohort is currently being investigated in a separate study.

Previous studies have suggested that inflammatory and infectious diseases might be associated with triggering the progression of MM and MGUS, indicating that a predisposition to infections could play a role in the pathogenesis of plasma cell malignancies [13–15]. Alternatively, the increased infection burden may reflect a gradual decline in immune function accompanying disease evolution [16]. The latter aligns with our observation that immunoparesis was more common in SMM individuals compared to MGUS and comparators. In the analysis adjusted for immunoparesis, the association between SMM and infection risk was attenuated and no longer statistically significant, suggesting that immunoparesis may contribute to the increased susceptibility to infections in SMM. However, since the risk estimates remained elevated, immunoparesis alone is unlikely to fully explain the observed findings and may instead serve as a marker of more advanced disease rather than a direct causal factor.

Given the ongoing investigation into early intervention for SMM, supported by promising results from several clinical trials, the optimal approach to clinical management remains uncertain. Consequently, identifying patients who may benefit from treatment is of critical importance [17–19]. Thus, our findings raise the important question of whether the individuals with SMM who experience more frequent infections are also of greater risk of progression to MM and may therefore benefit from early treatment. Furthermore, when considering treatment initiation in the high-risk SMM group, which may itself lead to treatment-related immunosuppression, it is essential to account for pre-existing infection risk. We believe that the findings of this study form a foundation for guidance regarding counseling individuals with SMM. The reported increased infection risk warrants heightened awareness of susceptibility to infections among individuals with SMM. Although the design of the study limits what can be concluded regarding e.g., immunization and prophylactic administration of antibiotics or immunoglobulins to SMM, we recommend exploring this in future studies, and that clinicians follow current guidelines for vaccinations for this group [20].

This is, to our knowledge, the first and only screened SMM cohort, providing a large population-based dataset with nationwide data. This design minimizes selection bias commonly seen in clinical cohorts and represents a major strength of the study. However, there are some limitations to this study. First, the subclassification of infections is difficult and might not be completely accurate as some infections can have several possible microbial causes, which could cause a skew in the distribution of infection subtypes. Furthermore, clinicians might tend to not register infections by ICD-10 codes along with prescribing antimicrobials, as is reflected in a 2.5 to 3-fold higher number of prescribed antimicrobials than ICD-10 coded infections in the cohort. However, this should affect SMM, MGUS and MGUS-free comparators equally. A potential surveillance bias could influence the results since the individuals with SMM in this study are all followed in the iStopMM study clinic, which could lead to more registered infections compared to the comparators. Furthermore, some of the viral infections may be underreported because they rarely lead to contact with primary care or hospitals and therefore are not registered. Finally, our cohort consists primarily of low-risk SMM, and therefore the results might not be transferrable to a clinical setting, with relatively more cases being in the intermediate or high-risk group. Future studies should consider analyzing infection risk in high-risk SMM to better characterize infection risk within a clinically representative cohort. However, in the present study, this was not feasible as many high-risk SMM patients from the iStopMM cohort are enrolled in the iStopMM treatment trial evaluating early treatment initiation and were therefore censored from the analysis early, limiting follow-up time for this group.

To summarize, in our population based screened SMM cohort we found that individuals with SMM have a significantly increased risk of infections compared to both individuals with MGUS and individuals without MGUS, a risk that was only partly explained by immunoparesis. Additionally, individuals with SMM received significantly more antibacterial prescriptions, suggesting a potentially larger bacterial infectious burden. These findings reveal an underrecognized vulnerability in SMM, even in a low-risk cohort. A better understanding of the underlying mechanisms may help identify patients who could benefit from preventive measures, enhanced surveillance, or early therapeutic intervention.

## Supplementary information

Supplemental material

## Data Availability

Current approvals do not allow for sharing of study data. Data may be shared on reasonable request pending the review of the investigators and the Icelandic National Bioethics Committee.
