# Supplementary material for: Increased risk of infections in smoldering multiple myeloma: results from the screened iStopMM study
Source: Leukemia. 2025 Sep 12;39(12):2997–3003. doi: 10.1038/s41375-025-02762-9 (PMC12634437; doi:10.1038/s41375-025-02762-9)
Supplement: Supplementary file 1 — Supplemental material [file 41375_2025_2762_MOESM1_ESM.docx]

# Supplementary

## ICD-10 and ATC-codes (Tables 1-4)

***Supplementary Table 1****: List of ICD 10-codes used to define infection events in the study. ICD-10 codes were categorized into bacterial, viral, fungal, and other categories based on the identified or presumed pathogen.*

| **Pathogen** | **ICD-10** |
| --- | --- |
| *Bacterial* | A00, A01, A02, A03, A04, A05, A15, A16, A17, A18, A19, A20, A200, A201, A202, A203, A207, A208, A209, A21, A210, A211, A212, A213, A217, A218, A219, A22, A220, A221, A222, A227, A228, A229, A23, A24, A25, A26, A27, A28, A30, A31, A310, A311, A318, A319, A32, A320, A321, A327, A328, A329, A33, A34, A35, A36, A360, A361, A362, A363, A368, A369, A37, A38, A39, A390, A391, A392, A393, A394, A395, A398, A399, A40, A400, A401, A402, A403, A408, A409, A41, A410, A411, A412, A413, A414, A415, A418, A419, A42, A420, A421, A422, A427, A428, A429, A43, A430, A431, A438, A439, A44, A440, A441, A448, A449, A46, A48, A480, A481, A482, A483, A484, A488, A49, A490, A491, A492, A493, A498, A499, A51, A52, A53, A54, A55, A56, A57, A58, A65, A66, A67, A68, A69, A70, A71, A74, A75, A77, A78, A79, B95, B950, B951, B952, B953, B954, B955, B956, B957, B958, B96, B960, B961, B962, B963, B964, B965, B966, B967, B968, B98, B980, B981, E321, G00, G000, G001, G002, G003, G008, G009, G01, G042, G050, H100, H440, H600, H601, H603, H609, H610, H620, H650, H66, H670, H70, I00, I01, I320, I33, I38, I39, I410, I520, I980, J01, J02, J020, J03, J030, J13, J14, J15, J170, J200, J201, J202, J22, J340, J36, J390, J391, J440, J65, J85, J86, K046, K047, K113, K122, K35, K570, K572, K574, K578, K61, K630, K670, K671, K672, K673, K750, K803, K804, K81, K830, L00, L01, L02, L03, L050, M000, M001, M002, M008, M010, M011, M012, M013, M462, M463, M630, M650, M710, M726, M86, M900, N151, N30, N390, N740, N741, N742, N743, N744, O740, O85, O883, O91, R572, T802, T814, T826, T827, T835, T836, T845, T846, T847, T857, T880 |
| *Fungal* | B35, B36, B37, B370, B371, B372, B373, B374, B375, B376, B377, B378, B379, B38, B380, B381, B382, B383, B384, B387, B388, B389, B39, B390, B391, B392, B393, B394, B395, B399, B40, B400, B401, B402, B403, B407, B408, B409, B41, B42, B420, B421, B427, B428, B429, B43, B430, B431, B432, B438, B439, B44, B440, B441, B442, B447, B448, B449, B45, B450, B451, B452, B453, B457, B458, B459, B46, B460, B461, B462, B463, B464, B465, B468, B469, B47, B48, B480, B481, B482, B483, B484, B485, B487, B488, B49, G021, H622, J172, M016 |
| *Viral* | A08, A60, A80, A81, A82, A83, A84, A85, A86, A87, A88, A89, A92, A93, A94, A95, A96, A97, A98, A99, B00, B000, B001, B002, B003, B004, B005, B007, B008, B009, B01, B010, B011, B012, B018, B019, B02, B020, B021, B022, B023, B027, B028, B029, B03, B04, B05, B050, B051, B052, B053, B054, B058, B059, B06, B060, B068, B069, B07, B08, B09, B15, B16, B17, B18, B19, B20, B21, B22, B23, B24, B25, B250, B251, B252, B258, B259, B26, B260, B261, B262, B263, B268, B269, B27, B30, B33, B34, B340, B341, B342, B343, B344, B348, B349, B97, B970, B971, B972, B973, B974, B975, B976, B977, B978, G020, G041, G051, H191, H621, H671, I411, J09, J10, J11, J12, J171, J203, J204, J205, J206, J207, J209, J210, J211, M014, M015, U071 |
| *Parasitic* | A06, A07, A59, B50, B51, B52, B53, B54, B55, B56, B57, B58, B580, B581, B582, B583, B588, B589, B60, B64, B65, B67, B68, B69, B690, B691, B698, B699, B70, B71, B72, B73, B74, B75, B76, B77, B78, B79, B80, B81, B82, B83, B85, B86, B87, B88, B89, H130, J173, M631 |
| *Other* | A09, A63, A64, B66, B90, B900, B901, B902, B908, B909, B91, B92, B94, B940, B941, B942, B948, B949, B99, E060, G02, G028, G03, G04, G040, G048, G049, G05, G052, G06, G07, G08, H000, H010, H03, H050, H105, H131, H190, H192, H320, H451, H481, H623, H624, H730, H731, H750, H830, H940, I301, I321, I400, I412, I430, I521, I681, I88, I981, J00, J04, J05, J06, J16, J17, J18, J20, J208, J21, J212, J31, J32, J80, K050, K051, K052, K053, K112, K140, K20, K65, K67, K678, K871, L04, L08, M00, M009, M01, M018, M464, M465, M60, M632, M651, M711, M901, M902, N080, N136, N159, N160, N288, N291, N34, N41, N431, N45, N481, N482, N49, N51, N61, N70, N71, N72, N73, N74, N748, N75, N76, N77, N980, O080, O23, O411, O86, O98, R650, R651 |

***Supplementary Table 2****: List of ATC-codes of antimicrobial prescriptions used to define infection events in the study. ATC-codes were categorized by therapeutic group (antibacterial, antiviral, and antifungal) based on infection type.*

| **Category** | **ATC code** |
| --- | --- |
| *Antibacterial* | A07AA09, A07AA11, J01AA01, J01AA02, J01AA04, J01AA07, J01AA08, J01AA12, J01CA01, J01CA02, J01CA04, J01CA08, J01CA12, J01CE01, J01CE02, J01CE08, J01CF01, J01CF02, J01CF05, J01CR02, J01CR05, J01DA01, J01DA06, J01DA23, J01DA33, J01DB01, J01DB04, J01DC02, J01DD, J01DD01, J01DD02, J01DD04, J01DF01, J01DH02, J01DH03, J01DH51, J01EA01, J01EC02, J01EE01, J01FA01, J01FA06, J01FA09, J01FA10, J01FF01, J01GB01, J01GB03, J01GB05, J01GB06, J01MA01, J01MA02, J01MA14, J01XA01, J01XB01, J01XD01, J01XE01, J01XX01, J01XX05, J01XX08, J01XX09, J04AB02, J04AB04, J04AC01, J04AK01, J04AK02, J04BA01, J04BA02, P01AB01 |
| *Antimycotic* | A07AA02, D01BA02, J02AA01, J02AB02, J02AC01, J02AC02, J02AC03, J02AC04, J02AX01, J02AX05, J02AX06 |
| *Antiviral* | J05AB01, J05AB04, J05AB06, J05AB09, J05AB11, J05AB12, J05AB14, J05AD01, J05AE01, J05AE03, J05AE07, J05AE08, J05AE10, J05AE11, J05AE12, J05AF05, J05AF07, J05AF09, J05AF10, J05AG01, J05AG03, J05AG04, J05AG05, J05AH01, J05AH02, J05AP01, J05AP08, J05AP51, J05AP55, J05AR, J05AR01, J05AR02, J05AR03, J05AR04, J05AR06, J05AR08, J05AR09, J05AR10, J05AR13, J05AR17, J05AR18, J05AR19, J05AR20, J05AX, J05AX08, J05AX12, J05AX1 5, J05AX27 |

***Supplementary Table 3:*** *Table of ICD-10 codes and corresponding infection diagnoses recorded during the study period, presenting the number of occurrences among individuals SMM, MGUS and MGUS-free comparators.*

*Viral infections*

| **ICD-10 Code** | **Infection diagnosis** | **MGUS-free comparators** | **SMM**  (matched with MGUS-free comparators) | **MGUS** | **SMM**  (matched with MGUS individuals) |
| --- | --- | --- | --- | --- | --- |
| B001 | Herpes simplex infection | 1 | 0 | 0 | 0 |
| B009 | Herpes simplex infection, unspecified | 5 | 0 | 0 | 0 |
| B02 | Shingles | 16 | 4 | 2 | 4 |
| B022 | Shingles with other complications | 3 | 0 | 0 | 0 |
| B029 | Shingles, unspecified | 6 | 0 | 1 | 0 |
| B07 | Warts | 13 | 3 | 5 | 4 |
| B26 | Mumps | 2 | 1 | 0 | 2 |
| B342 | Other specified viral infection | 1 | 0 | 0 | 0 |
| B349 | Viral infection, unspecified | 103 | 14 | 17 | 16 |
| H191 | Keratoconjunctivitis | 1 | 0 | 0 | 0 |
| J11 | Influenza, virus not identified | 1 | 0 | 0 | 0 |
| J209 | Acute bronchitis, unspecified | 77 | 20 | 15 | 18 |
| U071 | COVID-19, virus identified | 26 | 8 | 7 | 9 |
| B028 | Shingles with other complications | 0 | 0 | 2 | 0 |

*Bacterial infections*

| **ICD-10 Code** | **Infection diagnosis** | **MGUS-free comparators** | **SMM**  (matched with MGUS-free comparators) | **MGUS** | **SMM**  (matched with MGUS individuals) |
| --- | --- | --- | --- | --- | --- |
| A38 | Scarlet fever | 1 | 0 | 0 | 0 |
| A46 | Erysipelas | 10 | 2 | 3 | 2 |
| A49 | Bacterial infection, unspecified | 0 | 1 | 0 | 1 |
| A498 | Other specified bacterial infection | 0 | 1 | 0 | 1 |
| A499 | Bacterial infection, unspecified | 1 | 0 | 0 | 0 |
| B980 | Helicobacter pylori infection | 1 | 1 | 0 | 0 |
| H100 | Acute conjunctivitis | 2 | 0 | 0 | 0 |
| H601 | Acute otitis externa | 1 | 0 | 0 | 0 |
| H609 | Otitis externa, unspecified | 4 | 0 | 2 | 1 |
| H610 | Perichondritis auriculae | 1 | 0 | 0 | 0 |
| H650 | Acute otitis media | 1 | 1 | 0 | 0 |
| I38 | Endocarditis | 2 | 0 | 0 | 0 |
| J01 | Acute sinusitis | 101 | 13 | 15 | 12 |
| J020 | Acute streptococcal pharyngitis | 4 | 1 | 0 | 1 |
| J03 | Acute tonsillitis | 3 | 3 | 0 | 3 |
| J030 | Acute streptococcal tonsillitis | 2 | 0 | 0 | 0 |
| J15 | Bacterial pneumonia | 1 | 0 | 0 | 0 |
| J22 | Acute respiratory infection, unspecified | 19 | 6 | 3 | 7 |
| J340 | Nasal polyp | 1 | 0 | 0 | 0 |
| J440 | Chronic bronchitis | 5 | 0 | 0 | 0 |
| K047 | Periapical abscess without fistula | 2 | 0 | 0 | 0 |
| K35 | Acute appendicitis | 1 | 0 | 0 | 0 |
| K572 | Chronic enteritis | 2 | 0 | 0 | 0 |
| K578 | Other specified enteritis | 12 | 5 | 1 | 5 |
| K804 | Acute cholecystitis | 1 | 0 | 0 | 0 |
| K830 | Cholangitis | 4 | 0 | 0 | 0 |
| L01 | Impetigo | 9 | 3 | 1 | 4 |
| L02 | Skin abscess | 7 | 1 | 1 | 1 |
| L03 | Cellulitis | 25 | 3 | 4 | 3 |
| M710 | Bursitis | 1 | 0 | 0 | 0 |
| M86 | Osteomyelitis | 2 | 0 | 0 | 0 |
| N30 | Cystitis | 36 | 2 | 11 | 2 |
| N390 | Urinary tract infection, unspecified | 70 | 13 | 14 | 13 |
| T814 | Infection following medical procedure | 1 | 3 | 1 | 3 |
| T845 | Infection and inflammation due to prosthesis | 0 | 1 | 0 | 1 |
| B965 | Helicobacter pylori infection | 0 | 0 | 1 | 0 |

*Fungal infections*

| **ICD-10 Code** | **Infection diagnosis** | **MGUS-free comparators** | **SMM**  (matched with MGUS-free comparators) | **MGUS** | **SMM**  (matched with MGUS individuals) |
| --- | --- | --- | --- | --- | --- |
| B35 | Dermatophytosis | 43 | 23 | 8 | 22 |
| B37 | Candidiasis | 3 | 0 | 0 | 0 |
| B370 | Oral candidiasis | 6 | 0 | 4 | 0 |
| B372 | Candidiasis of skin and nails | 5 | 0 | 1 | 0 |
| B373 | Candidiasis of vulva and vagina | 7 | 0 | 1 | 0 |
| B374 | Candidiasis of other sites | 2 | 0 | 0 | 0 |
| B379 | Candidiasis, unspecified | 5 | 1 | 1 | 1 |

*Other infections*

| **ICD-10 Code** | **Infection diagnosis** | **MGUS-free comparators** | **SMM**  (matched with MGUS-free comparators) | **MGUS** | **SMM**  (matched with MGUS individuals) |
| --- | --- | --- | --- | --- | --- |
| A09 | Diarrhea and gastroenteritis of infectious origin | 13 | 5 | 1 | 4 |
| B91 | Sequelae of poliomyelitis | 3 | 4 | 1 | 4 |
| B94 | Sequelae of other infectious diseases | 1 | 0 | 0 | 0 |
| H000 | Hordeolum and other dyshidrotic conditions | 10 | 4 | 4 | 2 |
| H010 | Blepharitis | 15 | 3 | 7 | 4 |
| H050 | Acute inflammation of orbit | 0 | 1 | 0 | 1 |
| H105 | Acute dacryoadenitis | 3 | 2 | 0 | 2 |
| J00 | Acute nasopharyngitis | 50 | 8 | 13 | 7 |
| J06 | Acute upper respiratory infection | 1 | 0 | 0 | 0 |
| J18 | Pneumonia, unspecified | 3 | 0 | 0 | 0 |
| J20 | Acute bronchitis | 37 | 13 | 11 | 12 |
| J21 | Acute bronchiolitis | 0 | 2 | 0 | 2 |
| J31 | Chronic rhinitis | 4 | 0 | 0 | 0 |
| J32 | Chronic sinusitis | 28 | 1 | 1 | 1 |
| K050 | Acute gingivitis | 2 | 0 | 0 | 0 |
| K112 | Sialoadenitis | 1 | 0 | 0 | 0 |
| K140 | Glossitis | 1 | 0 | 0 | 0 |
| K20 | Esophagitis | 12 | 1 | 0 | 1 |
| M60 | Myositis | 17 | 1 | 3 | 1 |
| M711 | Bursitis | 0 | 1 | 0 | 1 |
| N34 | Urethritis | 2 | 0 | 0 | 0 |
| N45 | Orchitis | 2 | 0 | 0 | 0 |
| N481 | Balanitis | 7 | 1 | 0 | 1 |
| N76 | Vaginitis | 2 | 0 | 0 | 0 |
| B90 | Sequelae of tuberculosis | 0 | 0 | 1 | 0 |
| J04 | Acute laryngitis | 0 | 0 | 1 | 0 |
| K65 | Peritonitis | 0 | 0 | 2 | 0 |
| M464 | Ankylosing spondylitis | 0 | 0 | 2 | 0 |

***Supplementary Table 4:*** *Table of ATC codes and corresponding antimicrobial agent recorded during the study period, presenting the number of prescriptions among individuals SMM, MGUS and MGUS-free comparators.*

*Antibacterials*

| **ATC Code** | **Antimicrobial agent** | **MGUS-free comparators** | **SMM**  (matched with MGUS-free comparators) | **MGUS** | **SMM**  (matched with MGUS individuals) |
| --- | --- | --- | --- | --- | --- |
| A07AA11 | Nystatin | 1 | 0 | 0 | 0 |
| J01AA02 | Doxycycline | 160 | 40 | 46 | 29 |
| J01AA04 | Tetracycline | 4 | 0 | 1 | 0 |
| J01CA01 | Ampicillin | 8 | 1 | 2 | 1 |
| J01CA04 | Amoxicillin | 269 | 61 | 66 | 58 |
| J01CA08 | Pivampicillin | 277 | 39 | 57 | 42 |
| J01CE01 | Phenoxymethylpenicillin | 3 | 0 | 1 | 0 |
| J01CE02 | Benzylpenicillin | 99 | 17 | 16 | 17 |
| J01CF01 | Methicillin | 177 | 40 | 27 | 39 |
| J01CF02 | Oxacillin | 34 | 4 | 6 | 4 |
| J01CR02 | Amoxicillin and clavulanic acid | 290 | 48 | 48 | 53 |
| J01CR05 | Piperacillin and tazobactam | 4 | 0 | 2 | 0 |
| J01DA01 | Cefalexin | 8 | 2 | 0 | 2 |
| J01DB01 | Cefadroxil | 78 | 19 | 22 | 20 |
| J01DB04 | Cefradine | 53 | 6 | 9 | 6 |
| J01DC02 | Cefuroxime | 9 | 2 | 2 | 2 |
| J01DD02 | Cefotaxime | 1 | 1 | 1 | 1 |
| J01DD04 | Ceftriaxone | 47 | 7 | 5 | 6 |
| J01DH02 | Imipenem and cilastatin | 1 | 0 | 0 | 0 |
| J01DH03 | Meropenem | 2 | 0 | 0 | 0 |
| J01EA01 | Fosfomycin | 62 | 17 | 10 | 16 |
| J01EE01 | Sulfamethoxazole and trimethoprim | 31 | 5 | 11 | 5 |
| J01FA01 | Erythromycin | 9 | 2 | 3 | 2 |
| J01FA09 | Clarithromycin | 30 | 7 | 11 | 6 |
| J01FA10 | Azithromycin | 168 | 44 | 32 | 45 |
| J01FF01 | Clindamycin | 35 | 12 | 4 | 11 |
| J01GB03 | Tobramycin | 10 | 1 | 3 | 1 |
| J01MA02 | Ciprofloxacin | 133 | 25 | 21 | 24 |
| J01XA01 | Vancomycin | 3 | 2 | 2 | 2 |
| J01XD01 | Nitrofurantoin | 29 | 2 | 2 | 1 |
| J01XE01 | Metronidazole | 73 | 7 | 7 | 7 |
| J01XX05 | Methenamine | 22 | 0 | 1 | 0 |
| P01AB01 | Metronidazole | 40 | 6 | 11 | 6 |
| A07AA09 | Neomycin | 0 | 0 | 3 | 0 |

*Antivirals*

| **ATC Code** | **Antimicrobial agent** | **MGUS-free comparators** | **SMM**  (matched with MGUS-free comparators) | **MGUS** | **SMM**  (matched with MGUS individuals) |
| --- | --- | --- | --- | --- | --- |
| J05AB01 | Aciclovir | 2 | 0 | 0 | 0 |
| J05AB11 | Valaciclovir | 71 | 11 | 35 | 11 |
| J05AH02 | Oseltamivir | 10 | 2 | 1 | 2 |

*Antifungals*

| **ATC Code** | **Antimicrobial agent** | **MGUS-free comparators** | **SMM**  (matched with MGUS-free comparators) | **MGUS** | **SMM**  (matched with MGUS individuals) |
| --- | --- | --- | --- | --- | --- |
| A07AA02 | Nystatin | 17 | 2 | 4 | 2 |
| D01BA02 | Terbinafine | 90 | 5 | 9 | 5 |
| J02AC01 | Fluconazole | 92 | 21 | 16 | 22 |
| J02AC02 | Itraconazole | 9 | 0 | 5 | 0 |
| J02AX05 | Micafungin | 1 | 0 | 0 | 0 |

***Supplementary Table 5.*** *Frequencies of the most common infections and antimicrobial prescriptions among individuals with SMM and MGUS-free comparators, presented separately as overall totals and stratified by subtype.*

|  | **SMM** | **MGUS-free comparators** |
| --- | --- | --- |
|  | Infections (N) | Infections (N) |
| **Overall** | Acute bronchitis (33)  Dermatophytosis (23)  Viral infection, uns. (14)  Acute sinusitis (13)  Urinary tract infection (13) | Acute bronchitis (114)  Viral infection, uns. (103)  Acute sinusitis (101)  Urinary tract infection (70)  Acute nasopharyngitis (50) |
| **Bacterial** | Acute sinusitis (13)  Urinary tract infection (13)  Acute lower respiratory infection uns. (6)  Diverticulitis (5)  Acute tonsilitis (3) | Acute sinusitis (101)  Urinary tract infection (70)  Cystitis (36)  Cellulitis (25)  Acute lower respiratory infection uns. (19) |
| **Viral** | Acute bronchitis (33)  Viral infection uns. (14)  Covid-19 (8)  Herpes zoster (4)  Viral warts (3) | Acute bronchitis (114)  Viral infection uns. (103)  Covid-19 (26)  Herpes zoster (16)  Viral warts (13) |
| **Fungal** | Dermatophytosis (23)  Candidiasis uns. (1) | Dermatophytosis (43)  Candidiasis of vulva and vagina (7)  Candida stomatitis (6)  Candidiasis uns. (5)  Candidiasis of skin and nail (5) |
| **Other** | Acute nasopharyngitis (8)  Gastroenteritis uns. (5)  Hordeolum (4)  Blepharitis (3) | Acute nasopharyngitis (50)  Chronic sinusitis (28)  Infectious myositis (17)  Blepharitis (15)  Gastroenteritis uns. (13) |
|  | Antimicrobials (N) | Antimicrobials (N) |
| **Overall** | Amoxicillin (61)  Amoxicillin/clavulanic acid (48)  Azithromycin (44)  Dicloxacillin (40)  Doxycyclin (40) | Amoxicillin/clavulanic acid (290)  Pivmecillinam (277)  Amoxicillin (269)  Dicloxacillin (177)  Azithromycin (168) |
| **Antibacterial** | Amoxicillin (61)  Amoxicillin/clavulanic acid (48)  Azithromycin (44)  Dicloxacillin (40)  Doxycyclin (40) | Amoxicillin/clavulanic acid (290)  Pivmecillinam (277)  Amoxicillin (269)  Dicloxacillin (177)  Azithromycin (168) |
| **Antiviral** | Valaciclovir (11)  Oseltamivir (2) | Valaciclovir (71)  Oseltamivir (10)  Aciclovir (2) |
| **Antifungal** | Fluconazol (21)  Terbinafin (5)  Nystatin (2) | Fluconazol (92)  Terbinafin (90)  Nystatin (17)  Itraconazol (9) |

***Supplementary Table 6.*** *Frequencies of the most common infections and antimicrobial prescriptions among individuals with SMM and individuals with MGUS, presented separately as overall totals and stratified by subtype.*

|  | **SMM** | **MGUS** |
| --- | --- | --- |
|  | Infections (N) | Infections (N) |
| **Overall** | Acute bronchitis (30)  Dermatophytosis (22)  Viral infection, uns. (16)  Urinary tract infection (13)  Acute sinusitis (12) | Acute bronchitis (26)  Viral infection, uns. (17)  Acute sinusitis (15)  Urinary tract infection (14)  Acute nasopharyngitis (13) |
| **Bacterial** | Urinary tract infection (13)  Acute sinusitis (12)  Acute lower respiratory infection uns. (7)  Diverticulitis (5)  Impetigo (4) | Acute sinusitis (15)  Urinary tract infection (14)  Cystitis (11)  Cellulitis (4)  Acute lower respiratory infection uns. (3) |
| **Viral** | Acute bronchitis (30)  Viral infection, uns. (16)  Covid-19 (9)  Herpes Zoster (4)  Viral wart (4) | Acute bronchitis (26)  Viral infection, uns. (17)  Covid-19 (7)  Viral wart (5)  Herpes zoster (2) |
| **Fungal** | Dermatophytosis (22)  Candidiasis uns. (1) | Dermatophytosis (8)  Candida stomatitis (4)  Candidiasis, uns. (1)  Candidiasis of skin and nail (1)  Candidiasis of vulva and vagina (1) |
| **Other** | Acute nasopharyngitis (7)  Gastroenteritis uns. (4)  Blepharitis (4)  Acute bronchiolitis (2) | Acute nasopharyngitis (13)  Gastroenteritis uns. (7)  Hordeolum (4)  Infective myositis (3) |
|  | Antimicrobials (N) | Antimicrobials (N) |
| **Overall** | Amoxicillin (58)  Amoxicillin/clavulanic acid (53)  Azithromycin (45)  Pivmecillinam (42)  Dicloxacillin (39) | Amoxicillin (66)  Pivmecillinam (57)  Amoxicillin/clavulanic acid (48)  Doxycyclin (46)  Valaciclovir (35) |
| **Antibacterial** | Amoxicillin (58)  Amoxicillin/clavulanic acid (53)  Azithromycin (45)  Pivmecillinam (42)  Dicloxacillin (39) | Amoxicillin (66)  Pivmecillinam (57)  Amoxicillin/clavulanic acid (48)  Doxycyclin (46)  Azithromycin (32) |
| **Antiviral** | Valaciclovir (11)  Oseltamivir (2) | Valaciclovir (35)  Oseltamivir (1) |
| **Antifungal** | Fluconazol (22)  Terbinafin (5)  Nystatin (2) | Fluconazol (16)  Terbinafin (9)  Itraconazol (5)  Nystatin (4) |
